# Unusual presentation of hepatitis B serological markers in an Amerindian community of Venezuela with a majority of occult cases

**DOI:** 10.1186/1743-422X-8-527

**Published:** 2011-12-09

**Authors:** Nathalia E Cardona, Carmen L Loureiro, Domingo J Garzaro, María C Duarte, Daisy M García, Milian C Pacheco, Isabelle Chemin, Flor H Pujol

**Affiliations:** 1 Servicio Autónomo: Centro Amazónico para la Investigación y Control de enfermedades Tropicales, Simón Bolívar "CAICET", Puerto Ayacucho, Venezuela; 2 Laboratorio de Virología Molecular, Centro de Microbiología y Biología Celular, Instituto Venezolano de Investigaciones Científicas, Apdo 20632, Caracas 1020-A, Venezuela; 3 INSERM U1052 CRCL, Lyon I University, Villeurbanne, France

**Keywords:** Hepatitis B virus, Occult infection, Amerindians

## Abstract

**Background:**

Occult hepatitis B infection (OBI) is characterized by the presence of hepatitis B virus (HBV) DNA in the absence of HBsAg in the serum of patients. The aim of this study was to characterize HBV infection among a Piaroa community, an Amerindian group which exhibits significant evidence of exposure to HBV but relatively low presence of HBsAg, and to explore the presence of OBI in this population.

**Results:**

Of 150 sera, with 17% anti-HBc and 1.3% HBsAg prevalence, 70 were tested for the presence of HBV DNA. From these, 25 (36%) were found positive for HBV DNA by PCR in the core region. Two of these 25 sera were HBsAg positive, indicating an overt infection. Of the remaining 68 sera tested, 23 exhibited OBI. Of these, 13 were HBV DNA out of 25 anti-HBc positive (52%) and 10 HBV DNA positive, out of 43 anti-HBc negative (23%), with a statistical significance of *p *= 0.03. Viral DNA and HBsAg were present intermittently in follow up sera of 13 individuals. Sequence analysis in the core region of the amplified DNA products showed that all the strains belonged to HBV genotype F3. The OBI isolates displayed 96-100% nucleotide identity between them. One isolate exhibited the co-circulation of a wild type variant with a variant with a premature stop codon at the core protein, and a variant exhibiting a deletion of 28 amino acids.

**Conclusions:**

The frequency of OBI found in this Amerindian group warrants further studies in other communities exhibiting different degrees of HBV exposure.

## Background

Hepatitis B virus (HBV) infection is a significant health concern among Amerindians in the Americas with high exposure being documented in several Amerindian groups [1]. However, the prevalence of active HBV infection, defined as positivity for HBV surface antigen (HBsAg) is variable among different Amerindian communities, coexisting in the same geographic environment [2]. In a recent study in the Venezuelan Amazon, anti-HBc prevalence ranged from 17 to 70% [2].

Occult hepatitis B virus infection (OBI) is characterized by the presence of hepatitis B virus (HBV) DNA in the absence of HBV surface antigen (HBsAg) [3,4]. OBI can lead to severe chronic manifestations including hepatocellular carcinoma (HCC) [5,6]. OBI has not been studied thoroughly in Amerindian populations and could be present in Amerindian populations exhibiting evidence of exposure to HBV without high prevalence of active infection. Indeed, OBI has been already described in Mexican Amerindians [7]. The aim of this study was to characterize HBV infection among a Piaroa community, an Amerindian group which exhibits significant evidence of exposure to HBV but relatively low presence of HBsAg [2], and to explore the presence of OBI in this population.

## Results

**Figure 1:**
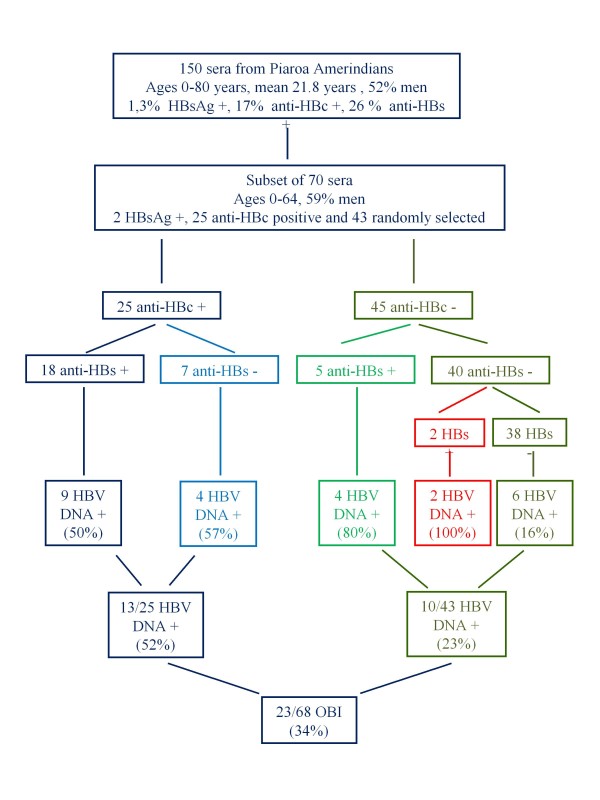
**HBV DNA detection according to the HBV serological profile in Piaroa Amerindians**.

A total of 150 sera from the Piaroa community Babilla de Pintao were analyzed (Figure 1). Total anticore antibodies (anti-HBc) prevalence was 17% (26/150) in this group and 31% (25/80) in individuals over 15 years of age [2]. Only 2 sera (1.3%) were positive for HBsAg [2]. These 2 sera were negative for anti-HBc antibodies. A subset of 70 sera was analyzed for the presence of HBV DNA. Of these, 25 (36%) were positive for HBV DNA by PCR in the core region (Figure 1). All individuals showed normal ALT levels. The 2 HBsAg sera were positive for HBV DNA. Of the remaining 23 sera, 13 were anti-HBc positive, and 10 were both anti-HBc and HBsAg negative. Among the HBsAg negative sera, 52% of the anti-HBc positive and 23% of the anti-HBc negative sera were HBV DNA positive, this difference being statistically significant (*p *= 0.03). HBV DNA was found even more frequently among anti-HBs positive individuals compared to anti-HBs negative ones (*p *= 0.01) (Figure 1). No difference was observed in the prevalence of OBI according to sex (9/25 of females and 16/41 of males had HBV DNA in their sera, *p *= 0.99), or to age (9/30 younger than 30 years vs. 12/25 older, *p *= 0.26).

Follow up sera were available for 13 individuals positive for HBV DNA. Viral DNA and HBsAg were present intermittently, as shown in Table 1. The two individuals presenting with an overt HBV infection at the beginning of the study, developed OBI later, since they carried HBV DNA in their sera for more than 2 years without the presence of HBsAg. The HBV genomic region that could readily be amplified was the core region, while the S region could be amplified only in some sera (Table 1). From the sera collected from vaccinated subjects in 2009, 34/36 showed levels of anti-HBs antibodies higher than 10 mIU/ml.

**Table 1:** HBV DNA in sera from Piarao Amerindians

Serum	Collected April 2002			Collected March 2003			Collected August 2004		
	**Serological status^1^**	**Core^2 ^DNA**	**S^2 ^DNA**	**Serological status^1^**	**Core^2 ^DNA**	**S^2 ^DNA**	**Serological status^1^**	**Core^2 ^DNA**	**S^2 ^DNA**

**BP131**	S +, AC -	+	-	S -, AC-	+	+	S +, AC -	+	-

**BP132**	S +, AC	+	-	S -, AC-	+	-	S -, AC+	+	-

**BP11**	S -, AC+	+							

**BP14**	S -, AC+	+							

**BP19**	S -, AC+	+					S -, AC+	+	+

**BP29**	S -, AC+	+	+						

**BP31**	S -, AC+	+							

**BP43**	S -, AC+	+							

**BP88**	S -, AC+	+	-						

**BP89**	S -, AC+	+							

**BP97**	S -, AC+	+	+	S -, AC+	+				

**BP113**	S -, AC+	+							

**BP117**	S -, AC+	+							

**BP152**	S -, AC+	+							

**BP168**	S -, AC+	+							

**BP21**	S -, AC-	+	-				S -, AC-	+	+

**BP74**	S -, AC-	+	-				S -, AC-	+	-

**BP92**	S -, AC-	+	-				S -, AC-	+	-

**BP134**	S -, AC-	+	-	S -, AC-	+				

**BP136**	S -, AC-	+	-	S -, AC-	+	-			

**BP147**	S -, AC-	+	+	S+, AC+	+	+	S -, AC+	+	-

**BP150**	S -, AC-	+	-						

**BP154**	S -, AC-	+	-	S -, AC-	+	-	S +, AC-	+	-

**BP156**	S -, AC-	+	-	S -, AC-	+	+	S -, AC-	+	-

**BP164**	S -, AC-	+	+	S +, AC-	+		S -, AC-	+	-

1: Serological status: S (HBsAg), AC (anti-HBc). 2: PCR of the core (C) or surface antigen (S) region. Blank cells mean not determined

**Figure 2:**
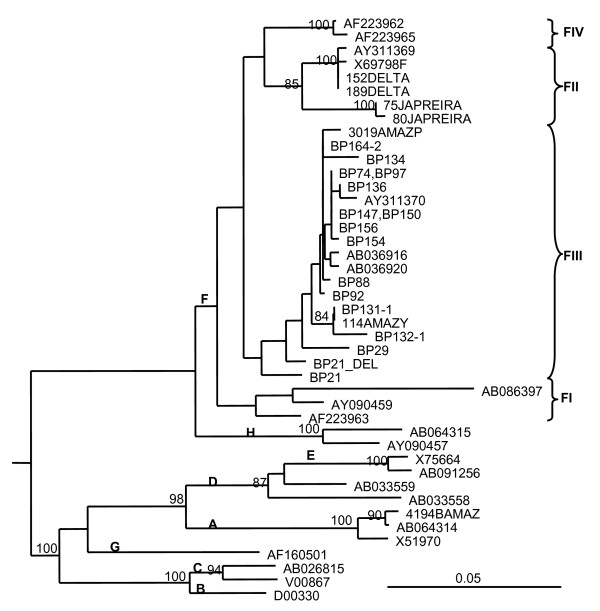
**Phylogenetic tree of HBV core gene region (438 nt)**. Isolates are designated by their GenBank accession number, except for Venezuelan isolates. Bootstrap values for the genotype and cluster branching are shown in the tree. Letters in bold indicate genotype and subgenotype. Venezuelan HBV isolates circulating in other Amerindians populations were included, from the Orinoco Delta (DELTA), in Yucpas (JAPREIRA) and in Yanomamis (Y) and Piaroas (P) from the Amazon (AMAZ)

**Figure 3:**
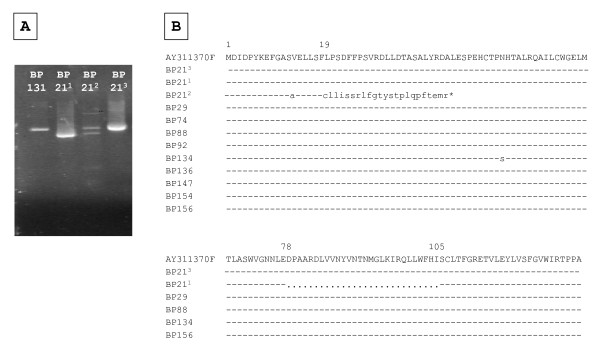
**HBV core variants circulating in a Piaroa Amerindian with OBI**. **a **Agarose gel electrophoresis of HBV core amplicons. *BP21 *repeated amplifications of isolate BP21 allow identifying several variants, each one more frequently found in one amplicon. *BP21^1 ^*variant carrying a deletion in the core region (309 nt). *BP21^2 ^*variant carrying a premature stop codon and a deletion. *BP21^3 ^*wild type variant (393 nt). **b **Amino acid alignments of the core region amplified in several isolates. Numbers indicate the aa position. Stop codon (*) and deletion (...) are shown.

Sequence analysis in the core region of the DNA amplified products showed that all the strains belonged to HBV genotype F3 (Figure 2). The OBI isolates displayed 96-100% nucleotide identity between them. The isolates were also closely related to sequences from HBV isolates circulating among other Piaroa, Yanomami and Yucpa Amerindians exhibiting overt infections and analyzed in previous studies [8]. One isolate, BP21, exhibited co-circulation of a wild type virus along with a variant harboring a premature stop codon at aa 42 of the core protein, and a variant exhibiting a deletion of 28 aas (aa 78-105) (Figure 3). A partial S genomic sequence was also available for 8 specimens. The sequences in the S region indicate the presence of HBV genotype F3, subtype adw4, although the length of the genomic region analyzed did not permit firm subgenotyping. Mutations associated with escape from antibody neutralization were not observed (data not shown). All the OBI strains were genetically related (Figure 2). Interestingly, 19/25 specimens of OBI shared at least one parent exhibiting OBI.

## Discussion

HBV DNA was analyzed in an Amerindian population exhibiting moderate prevalence of infection (17% anti-HBc), compared to other Venezuelan Amerindian populations, such as that of the Yanomami (58% anti-HBc). As described previously, this community showed a lower rate of acquisition of anti-HBc antibodies (1.4% in individuals less than 15 years old), compared to the Yanomami for example (38% in individuals less than 15 years old) [2]. The lower prevalence of HBV exposure and infection in this Amerindian community may be due to its geographic location, since being located near the main urban centre of the state, it is closer to health services. In addition, a more frequent contact with other civilizations may have modified some socio-cultural practices, leading to a reduction in HBV transmission, together with more effective accomplishment of vaccination programs. Despite the lower rate of HBV acquisition, this community still exhibited a 17% prevalence of anti-HBc antibodies, with a low prevalence of HBsAg positivity. OBI was shown in this Piaroa population, both in individuals with HBV serological markers and, with less frequency, in individuals with silent exposure to infection. Follow-up analysis in 15 individuals confirmed the presence of OBI, since HBV DNA could be detected in an intermittent form. The frequency of OBI in this community is higher than that found previously in blood donors from Caracas (4.3%) [9], and in Mexican and North American Amerindians (14.2% and 9.7% respectively) [7,10], although the methods used to determine OBI are somehow different between these reports. OBI is common among immunosuppressed individuals, due either to HIV [11,12], or to other causes [13]. It is important to note that Amerindians may be immunologically compromised due to multiple parasitic and bacterial infections, to add to the high prevalence of HBV exposure [14]. As expected, the prevalence of OBI infection was also higher when HBV serological markers of previous exposure (anti-HBc and/or anti-anti-HBs) were present. In addition, this Piaroa population exhibited a good response to vaccination as evidenced by the high frequency of seroconversion observed in 2009, after vaccination.

As anticipated, phylogenetic analysis showed the presence of the HBV genotype F3, and no particular strain was shown to be associated with OBI pattern, since the isolates were closely related to HBV isolates circulating in other Piaroa and Yanomami Venezuelan individuals [8]. In a previous study of Venezuelan blood donors, OBI was significantly associated with a higher prevalence of genotypes A and D (70%), while genotype F was predominant in overt cases (76%) [9]. The present study shows that OBI can also be very frequent among individuals exclusively exposed to HBV genotype F. OBI has been described recently in Nahuas and Huichol native populations from Mexico, and HBV genotype H was found in several cases [7]. Three studies have reported a predominance of genotype A and particularly D in cases of OBI [15-17], while in other studies, genotype A was present at a similar prevalence in overt and OBI infections [18]. Altogether, these studies suggest that OBI appears not to be restricted to a particular genotype. In our study, one subject was infected by a wild type virus with variants coding for core defective proteins, a situation already described in Venezuelan blood donors with OBI [9]. Most of the subjects with OBI were related, suggesting than familiar transmission might have played a role in this situation. However, the number of samples analyzed and the short genomic sequence available for study did not allow testing of this hypothesis.

There is accumulating evidence of a pathogenic role for OBI [19]. OBI may contribute to the progression of liver fibrosis and HCC development [20], thus the potential benefits of antiviral treatment is in debate [6,21]. As shown in this study and in others, vaccination of those populations at risk for OBI should be undertaken as it may bring some benefits to these communities [22].

## Conclusions

A high frequency of unusual HBV presentation was found in this Piaroa population. All the individuals were infected with HBV genotype F3. The OBI isolates displayed a restrained variability and were similar to the isolates causing overt HBV infection in other Venezuelan Amerindian groups. The frequency of OBI found in this Piaroa population warrants further studies in other Amerindian communities exhibiting different degrees of HBV exposure.

## Methods

### Population group

The Piaroa community of Babilla de Pintao (Amazon State, Venezuela) consists of 169 inhabitants, and 150 sera were analyzed for the presence of HBV serological markers, with informed consent and under approval of Bioethical Committees of CAICET and IVIC [2]. Individuals were also vaccinated during this period. Testing was performed between 2002 and 2004, and a subset of sera (n = 36) were collected in 2009 to evaluate anti-HBs antibodies.

### Serological assays

Sera were tested for HBV markers with commercial assays: HBcAb DIMA™ (DIMA Diagnostika C.A., Venezuela), Murex HBsAg Version 3 (ABBOTT, Murex Biotech Limited, UK), Bioelisa anti-HBs (Biokit, S.A., Spain) and IgM anti-HBc by ETI-CORE-IGMK-2 (DiaSorin Ltda., Italy). A sample was considered anti-HBs positive if the levels of anti-HBs antibodies were higher than 10 UI/ml. ALT were also determined with a commercial assay (Wiener Lab, Argentina).

### PCR and sequencing

A total of 70 sera (2 HBsAg positive, 25 anti-HBc positive and 43 remaining randomly selected) were analyzed by nested PCR of the core region [9]. A sample was considered positive if it repeated positive after a second extraction of viral DNA. When enough serum was available, samples were also amplified by nested PCR in the S region [8]. Purified PCR fragments were sent to CESAAN (Centro de Secuenciación y Análisis de Acidos Nucleicos, IVIC, Caracas, Venezuela), for sequencing. Sequences obtained from the Venezuelan isolates were compared with different reference strains from GenBank and used for phylogenetic analysis. Sequence alignment and phylogenetic analysis by the Neighbor Joining method (1,000 bootstrap replicas, genetic distances evaluated with Kimura 2 parameters corrections) were conducted using DNAMAN 5.2.2 (Lynnon Bio Soft, Canada). Nucleotide sequence data have been deposited in GenBank database under the accession numbers JN255220-JN255243.

### Statistical analysis

Statistical differences were evaluated by the Chi-Squares test with Yates correction, according to a computerized Epi Info program, version 3.3.2 (Centers for Disease Control and Prevention, Atlanta, GA).

## List of abbreviations

HBV: Hepatitis B virus; OBI: Occult hepatitis B virus infection; HCC: Hepatocellular carcinoma; HBsAg: HBV surface antigen; anti-HBc: Anticore antibodies

## Competing interests

The authors declare that they have no competing interests.

## Authors' contributions

NEC, CLL and DJG carried out the molecular genetic studies, and participated in the sequence alignment. NEC, IC and FHP drafted the manuscript. DMG and MCP carried out the immunoassays. MCD participated in the clinical and epidemiological study. NEC and FHP participated in the design of the study and performed the statistical analysis. All authors read and approved the final manuscript.
